# Single organic electrochemical neuron capable of anticoincidence detection

**DOI:** 10.1126/sciadv.adv3194

**Published:** 2025-06-20

**Authors:** Padinhare Cholakkal Harikesh, Dace Gao, Han-Yan Wu, Chi-Yuan Yang, Deyu Tu, Simone Fabiano

**Affiliations:** Laboratory of Organic Electronics, Department of Science and Technology, Linköping University, Norrköping, Sweden.

## Abstract

Emulating complex neural computations like solving linearly inseparable tasks within single artificial neurons has remained an elusive goal in neuromorphic engineering. Here, we report a dendritic organic electrochemical neuron (d-OECN) capable of achieving anticoincidence detection by classifying the exclusive-OR (XOR) problem—a quintessential linearly inseparable task—within an individual neuron. Inspired by human cortical neurons that perform XOR through dendritic calcium spikes, the d-OECN leverages ion-tunable antiambipolarity in mixed ionic-electronic conducting polymers to mimic voltage-gated dendritic calcium dynamics. By integrating this dendritic component with a tunable spiking circuit representing the soma, the d-OECN achieves XOR classification through its inherent nonlinear activation profile, with decision boundaries that are both ionically and electrically tunable. Moreover, we demonstrate the d-OECN’s ability to perform edge detection using XOR in a tactile sensing system, showcasing its potential for event-based sensing and processing. The d-OECNs, replicating key aspects of biological intelligence, pave the way for next-generation bioelectronics and robotics requiring complex neural computation.

## Teaser

A bioinspired artificial neuron solves complex XOR tasks, enabling edge detection for advanced bioelectronics and robotics.

## INTRODUCTION

Leveraging neuromorphic sensing and processing, inspired by the intricate workings of biological systems, holds great potential for the development of cutting-edge bioelectronic and robotic systems (*1*–*3*). Central to these neuromorphic systems are artificial neurons and synapses (*4*). For these artificial neural circuits to be effective and biointegrable, they require a design that is both simple and operates on ion-based mechanisms, mirroring biological processes. Unfortunately, neuromorphic circuits made from silicon and other inorganic materials fall short in these areas (*5*).

Organic semiconductor–based technologies, particularly those using organic electrochemical transistors (OECTs), which work on the basis of ion-based mechanisms, provide a promising solution to these limitations, as evidenced by their growing applications in artificial neurons (*6*–*9*), synapses (*10*–*15*), and neural interfaces (*16*–*19*). Neuromorphic circuits featuring OECT-based synapses have been used in a variety of applications, including nonlinear classification (*10*), sensorimotor integration and learning for robotics (*20*), retrainable neuromorphic biosensors with on-chip learning and classification capabilities (*15*), and biologically interfaced pattern classifiers (*21*). The recently developed organic electrochemical neurons (OECNs) (*6*, *7*, *22*) mark a further step forward by emulating key biological neural features, including neurotransmitter and ion-based modulation, that traditional silicon-based neurons cannot achieve. Despite these advancements, OECNs and artificial neurons based on alternative technologies (*23*–*29*), including conventional silicon-based technologies (*30*), still fall short of fully replicating the complete spectrum of biological neural functionalities. A critical and unresolved challenge for artificial neurons is their inability to classify linearly inseparable data, such as the exclusive-OR (XOR) function, within a single neuron device.

XOR (also known as anticoincidence detection) outputs true only when its two binary inputs differ. Unlike AND, OR, or NOT operations, which are linearly separable, XOR is inherently nonlinear and cannot be solved by a simple linear summation of inputs. This makes XOR a critical benchmark for testing nonlinearity in artificial neural networks. Historically, the XOR problem has played a pivotal role in shaping neural network architectures. In the 1960s, Minsky and Papert’s demonstration of single-layer perceptron’s inability to solve XOR (*31*) led to a marked decline in artificial intelligence research as it was believed that neural networks were ill-equipped to handle complex, real-world nonlinear problems. The subsequent introduction of multilayer networks and the backpropagation algorithm (*32*) in the 1980s revitalized artificial intelligence research, showcasing the potential of deeper architectures to overcome such limitations. However, this advancement came at the cost of increased complexity and computational demands, highlighting the ongoing need for more efficient solutions, particularly in hardware implementations where minimal circuit complexity is crucial, such as in edge computing or biologically interfaced devices. Now, XOR function mapping in artificial neural networks, including those using OECT-based circuits (*10*), still requires a multilayer neural network configuration, underscoring the challenge of achieving nonlinear activation functions in conventional single-compartment artificial neuron models.

**Fig. 1.**
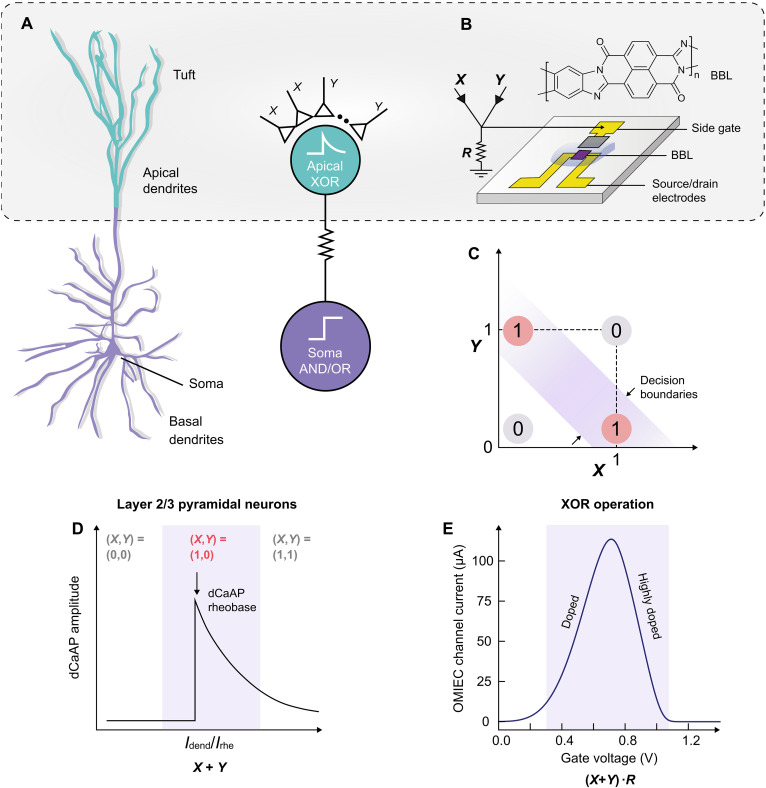
Implementation of anticoincidence detection with OECTs. (**A**) Structure of cortical layer 2/3 pyramidal neurons and their corresponding circuit representation. (**B**) OECT configuration designed to emulate the apical dendrite. (**C**) Graphical representation of XOR logic operation with multiple decision boundaries. (**D**) Relationship between the dCaAP amplitude and the input current to the dendrite. (**E**) Antiambipolar behavior demonstrated in BBL.

This contrasts with biological systems, where recent findings in neuroscience demonstrate that single human cortical neurons can address this challenge. Research has shown that layer 2 and layer 3 human cortical pyramidal neurons are capable of performing anticoincidence detection within their dendritic compartments using dendritic calcium spikes (*33*). The key to this operation lies in voltage-gated calcium channels in their dendritic compartments. These channels mediate local dependence where the amplitude peaks at a specific preferred input strength and declines for weaker or stronger inputs. This forms a Gaussian-tuned nonlinear activation unlike more linear relationships between input and output in conventional neurons. As a result, when two separate inputs of the preferred high amplitude (1,1) are applied simultaneously to different branches of the dendrite, the resulting sum of the inputs is too high, resulting in a diminished calcium spike amplitude (0). In contrast, when a preferred high input is applied to only one branch with no or low input to the other branch [corresponding to (1,0) or (0,1)], the sum is a preferred value that coincides with the peak of the Gaussian [dendritic calcium action potential (dCaAP) rheobase], resulting in a high final calcium spike amplitude (1). This implements a version of anticoincidence detection—the essence of XOR logic (Fig. 1).

Before and since these findings, various studies, including experimental (*34*, *35*) and mathematical models (*36*–*38*), have sought to emulate similar mechanisms for nonlinear computation in single neurons and neural networks. Neural network models like the radial basis function, probabilistic neural networks (*39*), and support vector machines (*40*) also use such nonlinear activation functions, although in a different manner than biological neurons, to improve learning capabilities. There have also been recent efforts to emulate the nonlinear dendritic behavior using sophisticated antiambipolar transistors made of two-dimensional chalcogenide heterojunctions to enhance the learning capabilities of spiking neural networks (*41*). However, hardware implementation of all these approaches, which are primarily based on inorganic materials, often requires intricate fabrication strategies to replicate the Gaussian activation profile and is not fully integrated with artificial spiking neurons. Moreover, their lack of responsiveness to ions and lack of biocompatibility limit their biointegration capabilities. The recently reported antiambipolar behavior in the n-type ladder polymer poly(benzimidazobenzophenanthroline) (BBL; Fig. 1E) is highly relevant in this context (*42*). Previously, we leveraged this behavior to simulate the ion channels of biological neurons (Na^+^ and K^+^), resulting in the creation of biorealistic conductance-based OECNs according to the Hodgkin-Huxley neural model (*6*). In this study, we use this phenomenon to mimic dCaAP-like functions generated by voltage-gated calcium channels found in dendrites (Fig. 1, D and E). These functions can be adjusted both ionically and electrically. By integrating with a spiking neuron model, we develop a dendritic organic electrochemical neuron (d-OECN), which is capable of classifying linearly inseparable data, such as the XOR problem, using a single neuron. This classification problem necessitates multiple decision boundaries and is beyond the capability of conventional single-layer perceptrons. Furthermore, we showcase the ability to modify the decision boundaries for such complex inputs through straightforward modulation of the neuron’s threshold voltages and ionic concentrations, an achievement not possible with single artificial neurons made from any other technology.

## RESULTS

### Dendritic calcium channel–integrated OECN for solving XOR

**Fig. 2.**
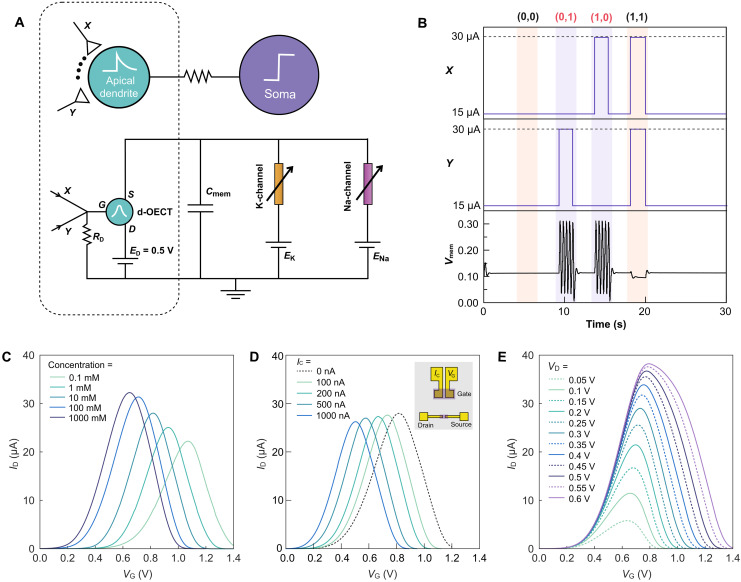
d-OECN and modulation of dCaAP behavior. (**A**) Artificial neuron circuit incorporating dendritic XOR functionality. The detailed circuit is available in fig. S1. (**B**) Spiking pattern of the neuron with various input combinations. (**C**) Tuning the peak position (mean) of the Gaussian function using the ionic concentration. (**D**) Adjusting the peak position of the Gaussian using a dual-gate configuration. (**E**) Modulating the standard deviation and amplitude of the Gaussian function using the drain voltage.

In Fig. 2A, the architecture of the d-OECN is divided into two integral components: the apical dendrite, responsible for executing nonlinear computations crucial for XOR logic functionality, and the soma, which implements a thresholding mechanism—triggering a spike only when the input stimuli exceed a specific threshold (*33*). To mimic the dendrite’s function, we use an antiambipolar BBL-based OECT, referred to as the dendritic OECT (d-OECT). The soma’s thresholding behavior is simulated using a Hodgkin-Huxley–based OECN, where the spiking characteristics are finely tuned by calibrating the potassium channel threshold. This calibration introduces an appropriate leakage current, ensuring the neuron spikes only when inputs surpass a predefined threshold (see fig. S1 and Supplementary Text). In addition, the dendritic compartment of the d-OECN can be integrated with a leaky integrate-and-fire OECN (*7*) functioning as the soma (figs. S2 to S4). This integration demonstrates the flexibility and adaptability of the approach across various neuron models, which also supports its potential for scalability.

After fine-tuning, we establish the d-OECN’s functional range for input currents (typically 2 to 15 μA). The d-OECT is then configured to deliver the required current range for the neuron to respond to preferred input combinations for XOR operations. These (*X*, *Y*) input pairs—here specifically (30 μA, 15 μA) and (15 μA, 30 μA)—result in spiking behavior, consistent with XOR logic. The (*X*, *Y*) currents are converted to voltages and summed at the d-OECT gate via a resistor (*R*_D_ = 18 kilohms; Fig. 2A). This configuration ensures that when a target (*X*, *Y*) combination for XOR logic is received, the neuron fires. Conversely, for inputs outside the XOR range, such as (15 μA, 15 μA) or (30 μA, 30 μA), the input voltage at the d-OECT falls at the extremes of the Gaussian curve, resulting in insufficient output drain current to induce a membrane voltage (*V*_mem_) spike (Fig. 2B). This mechanism effectively delineates the decision boundaries for XOR logic using the Gaussian function to distinguish between the linearly inseparable input combinations—mirroring the processing observed in layer 2/3 pyramidal neurons, as previously discussed. Notably, the d-OECN is capable of performing the XOR operation in response to 10-ms current inputs (fig. S5), a timescale comparable to that of biological neurons.

### Modulation of the dCaAP behavior in BBL

While we have showcased XOR functionality for a single combination of inputs *X* and *Y*, real-world applications require generalizing this functionality to classify a broader spectrum of inputs. Therefore, the tunability of the d-OECT is essential. Beyond the XOR application, the ability to adjust the mean, standard deviation, and amplitude of the Gaussian function is also advantageous for integrating d-OECTs into alternative neural network models like probabilistic neural networks (*43*). One method to modify the mean of the Gaussian function is to alter the ionic concentration of the electrolyte. Lowering the electrolyte concentration shifts the Gaussian peak toward higher gate voltages following a logarithmic relationship (Fig. 2C). This shift occurs because of voltage drops occurring at the gate/electrolyte and polymer/electrolyte interfaces, as described by Nernstian relationships (*6*, *44*) and in agreement with SPICE simulations of d-OECTs (fig. S6A). An alternative method is the electrical approach using a dual-gate configuration of OECTs (Fig. 2D, inset). In this setup, the gate comprises two gold electrodes connected by a BBL layer. Injecting a control current, *I*_C_, into this second gate results in an additional voltage equivalent to *I*_C_ multiplied by the resistance of the BBL layer on top of the original gate voltage *V*_G_. This adjustment shifts the peak position depending on the value of *I*_C_ injected, hence changing the mean of the Gaussian (see Fig. 2D and relative SPICE simulations in fig. S6B). It is also possible to alter both the amplitude and standard deviation of the Gaussian by varying the drain voltage of the d-OECT. Increased drain voltages lead to a Gaussian distribution with a wider full width at half maximum, attributed to varying doping levels at the drain and source electrodes (see Fig. 2E and relative SPICE simulations in fig. S6C).

### Modulating the decision boundaries

**Fig. 3.**
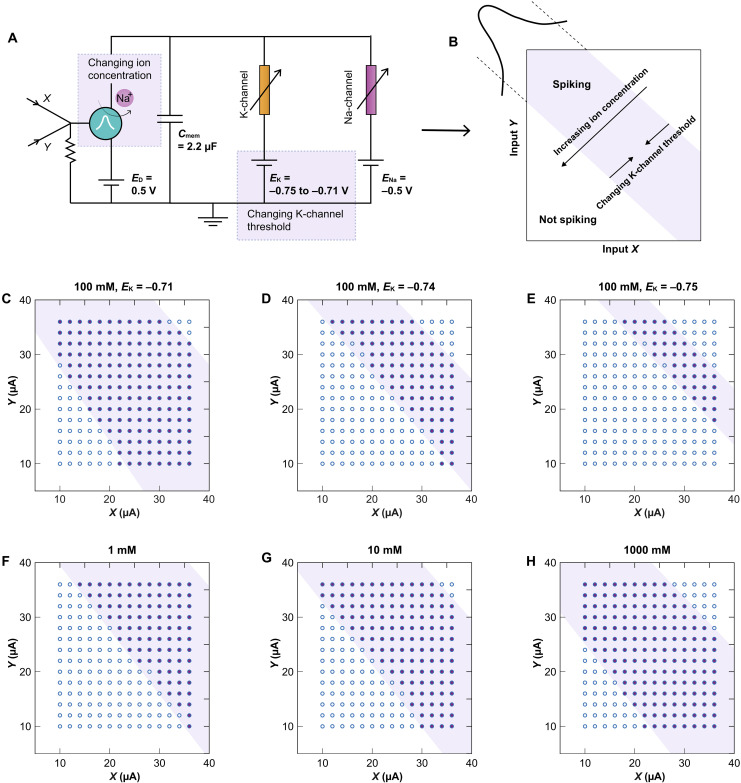
Modulation of the decision boundaries. (**A** and **B**) Artificial neuron circuit with the capability for XOR classification featuring tunable decision boundaries. The decision boundaries are delineated by the Gaussian function. (**C** to **E**) Narrowing the width of the decision boundary by adjusting the potassium channel threshold (electrolyte concentration, 100 mM). Solid dots represent combinations that trigger neuron spiking, while hollow dots represent nonspiking behavior. (**F** to **H**) Shifting the decision boundary by modifying the mean of the Gaussian through changes in ion concentration (*E*_K_ = −0.71 V). For all the measurements, the electrolyte is NaCl in water. The detailed circuit for the Na and K channels is provided in fig. S1.

The ability of d-OECN to process a range of (*X*, *Y*) input pairs for XOR tasks, rather than just discrete pairings, is essential for capturing the complexities of inputs encountered in real-world scenarios. To explore this capability, the d-OECN is exposed to a broad range of input currents, *X* and *Y*, to evaluate its response (Fig. 3A). The d-OECN is observed to spike in response to a continuum of (*X*, *Y*) inputs, rather than being limited to specific pairings. This behavior arises from the soma’s ability to respond to a wide range of input currents, where the input current is a combined function of *X* and *Y*. This enables a single d-OECN to perform XOR classification with functionality equivalent to a two-layer neural network (*45*). The tails of the Gaussian function define the decision boundaries. By altering the ion concentration, which shifts the mean of the d-OECT’s Gaussian response peak, the decision boundaries are repositioned accordingly [Fig. 3 (F to H) and relative SPICE simulation results in figs. S7 and S8]. In addition, the XOR functionality demonstrates resilience to device-to-device current variability in the d-OECT, with the upper decision boundary shifting by ∼6% for a current variability of around 10%, while the lower decision boundary remains unaffected (fig. S9).

The width of the decision region can also be fine-tuned by adjusting the K-channel threshold (Fig. 3, C to E). Shifting the K-channel threshold to more negative values allows additional input current to leak through that channel, thereby increasing the minimum input current required for the d-OECN to spike. The XOR decision boundaries can be dynamically modulated by injecting a control current into the secondary gate of the d-OECT (fig. S10). The use of a polarizable BBL gate in this configuration leads to instability in the transfer curves, which is partially mitigated by the application of an input current, as shown in figs. S15 and S16. In addition, integration with organic electrochemical synapses (*11*, *21*) offers additional modulation capabilities as the synaptic weights directly influence the slope of the decision boundaries for XOR classification (figs. S11 and S12). This extensive tunability in decision boundaries gives d-OECNs the flexibility to adapt to new patterns, akin to biological neurons that modify synaptic strengths during learning and memory. Such flexibility is vital during training, where the system refines its data classification, and equally important in real-world applications, where discerning and responding to a diverse array of stimuli are necessary. While full-scale integration into neural networks is beyond the scope of this work, in the following section, we present a preliminary example demonstrating how the d-OECN can simplify edge computing for biologically inspired applications, such as tactile edge detection.

### Simplifying edge detection in tactile sensing with the d-OECN

In biological systems, tactile edge detection arises from neurons responding to contrasts in mechanical input across their receptive fields. Mechanoreceptors, such as Merkel cells and Meissner corpuscles, play pivotal roles in this process. Merkel cells, which are slowly adapting (SA), respond to sustained pressure, while Meissner corpuscles, which are rapidly adapting (RA), detect dynamic changes in pressure (*46*). These mechanoreceptors work together to identify tactile features such as edges, where contrasting inputs—high pressure on one side and low pressure on the other—are detected by neighboring sensors. Tactile edge detection is further refined by lateral inhibition, a mechanism in which strongly activated neurons suppress the activity of neighboring neurons with similar input levels. This process sharpens contrasts and enhances the perception of boundaries, functioning in a manner analogous to XOR logic, which detects mismatched inputs through anticoincidence detection.

**Fig. 4.**
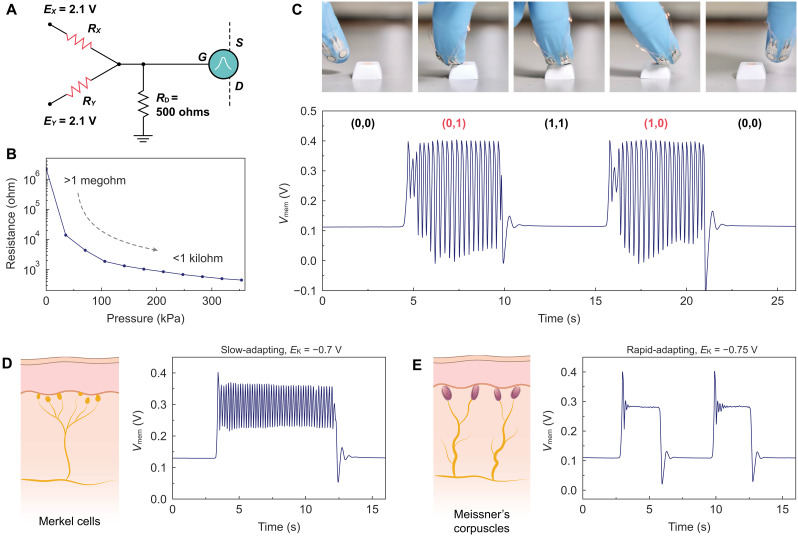
Edge detection and tactile sensing using d-OECN. (**A**) Circuit diagram illustrating the XOR-based edge detection setup with two resistive pressure sensors (*R_X_* and *R_Y_*) as inputs to the d-OECN. The neuron generates spikes only when one sensor is activated, demonstrating XOR operation. (**B**) Resistance change of the pressure sensor as a function of different pressure levels. (**C**) Experimental demonstration of edge detection: Sequential photos show the contact of two sensors with an object. The corresponding *V*_mem_ exhibits spiking behavior for XOR input conditions (0,1) and (1,0), but no spikes for non-XOR conditions (0,0) and (1,1). (**D**) Emulation of a slowly adapting Merkel-type mechanoreceptor: continuous spiking in response to sustained input pressure. (**E**) Emulation of a rapidly adapting Meissner-type mechanoreceptor: spiking in response to dynamic pressure changes. The mechanoreceptor functions are emulated by adjusting the potassium channel voltage (*E*_K_) in the Hodgkin-Huxley neuron model. With *E*_K_ at −0.7 V, a continuous, slow-adapting (class 1) spiking behavior is observed, similar to slowly adapting mechanoreceptors. Lowering *E*_K_ to −0.75 V produces a fast-adapting (class 3/phasic) response, where spiking occurs mainly at the onset of the input.

Inspired by these biological principles, we demonstrate the d-OECN’s ability to perform edge detection. The experimental setup, illustrated in Fig. 4A, consists of two resistive pressure sensors (*R_X_* and *R_Y_*, with pressure characteristics shown in fig. S13) connected to the d-OECN. These sensors generate input currents for the neuron on the basis of their resistance and the applied voltage (*E_X_*, *E_Y_*). When a resistive sensor is pressed against an object, its resistance decreases (Fig. 4B), resulting in an increase in current passing through the sensor. The circuit enables the d-OECN to execute an XOR operation, producing spikes only when one sensor is active (high pressure on one sensor and low pressure on the other). The d-OECN remains silent when both sensors are either inactive or simultaneously active, effectively replicating the principle of anticoincidence detection observed in biological systems.

In Fig. 4C, we validate the edge detection functionality of the d-OECN. Sequential contact of the sensors, mounted on a glove, with an object is recorded alongside the corresponding neuron’s *V*_mem_. The neuron exhibits spiking behavior exclusively under XOR conditions (0,1) or (1,0), while it remains silent under non-XOR conditions (0,0) or (1,1). When the finger is positioned entirely outside the object’s surface, both sensors are inactive, providing an input of (0,0) to the neuron. At the edge of the object, the input is (0,1) or (1,0), as one sensor contacts the surface while the other remains outside, satisfying the XOR condition. When both sensors are fully on the surface of the object, both inputs are active (1,1). This functionality allows the d-OECN to effectively detect the edge of the object.

In combination with XOR-based operations, the d-OECN can also emulate the behavior of biological mechanoreceptors, as demonstrated in Fig. 4 (D and E). When configured to mimic SA mechanoreceptors, such as Merkel cells, the d-OECN produces continuous spikes in response to sustained input pressure (Fig. 4D). Conversely, when configured to mimic RA mechanoreceptors, such as Meissner corpuscles, it generates spikes only during dynamic pressure changes (Fig. 4E) while simultaneously executing XOR-based edge detection. This reconfiguration is achieved by simply tuning the K-channel voltage of the soma compartment. These combined functionalities underscore the versatility of the d-OECN in real-time sensory processing.

## DISCUSSION

Historically, the XOR function, emblematic of linearly inseparable problems, has remained elusive for single-neuron models because of its demand for nonlinear activation functions. Our approach, inspired by the discovery of dendritic calcium spikes in human cortical neurons, uses a d-OECT modulated by ionic and electrical signals to emulate this biological behavior. By integrating dendritic calcium channel dynamics with an electrochemically tunable artificial soma, we have not only replicated a key feature of neuronal processing but also enabled the classification of XOR (anticoincidence detection) and other linearly inseparable inputs using a single-neuron setup. This advancement is pivotal, offering a simpler, more efficient pathway for neuromorphic design and biointegration, thus bringing artificial neurons a step closer to their biological counterparts.

While immediate applications of the XOR-capable d-OECNs may not be fully defined, it is common for the true potential of emerging technologies to unfold over time. One promising avenue could be enhancing the functionality of silicon-based neuromorphic processors for edge applications. OECNs are particularly suited for event-based sensing and processing applications because of their inherent ability to detect multiple sensory modalities, including ions, biomolecules, pressure, temperature, and light—capabilities not inherently present in traditional silicon-based neuronal circuits. Having the added ability to perform nonlinear classification could substantially enhance active processing at the sensor level with fewer neurons. This is demonstrated by our proof-of-concept implementation of edge detection in a tactile sensing system using a single neuron.

This capability holds strong relevance for applications such as artificial skin in robotics and prosthetics, where tactile edge detection allows systems to identify object boundaries, surface contours, and texture gradients—key for manipulation and feedback. Similarly, in neuromorphic vision systems, edge detection plays a central role in interpreting images. The anticoincidence detection behavior exhibited by the d-OECN is analogous to the function of lateral inhibition circuits found in the retina (*47*), which are responsible for enhancing edge contrast and sharpening visual perception. Our demonstration that a single d-OECN can detect tactile edges and dynamically reconfigure to mimic distinct mechanoreceptor behaviors provides a proof of concept for embedding such bioinspired computation directly at the sensory interface. This approach is particularly advantageous in event-based edge computing, where real-time, low-latency processing at the sensor level reduces the data load on downstream processors and supports efficient operation.

To realize these applications at scale, key technological challenges must be addressed, such as minimizing device footprint, increasing speed, reducing power consumption, and ensuring scalability to larger neural networks. In our system, power usage is primarily constrained by the operating speed and gate leakage of the OECTs, which govern spiking frequency and spike width. In the soma block of the d-OECN, ~80% of the total power (~50 μW of ~60 μW) is consumed by the inverter block, largely due to n-channel metal-oxide semiconductor amplifiers with resistive loads. This design, chosen to ensure system stability and desired voltage levels, could be optimized by replacing n-channel metal-oxide semiconductor inverters with complementary OECT inverters, which have demonstrated power consumption levels as low as ~1 μW (*48*–*50*). However, challenges such as stability and operating range currently hinder the adoption of these inverters. The dendritic block consumes comparatively less power (~5 μW), driven by the dendritic OECT drain voltage (0.3 to 0.5 V) and a maximum current of ~10 μA. This sufficiently high current helps minimize the impact of gate leakage currents, which are currently in the range of hundreds of nanoamperes. Reducing gate leakage by an order of magnitude could proportionally lower both input current requirements and overall power consumption. Further improvements, such as scaling down the OECT channel dimensions to reduce parasitic capacitances, could enhance firing frequencies and improve energy efficiency, addressing key performance requirements for practical implementation in neural networks. Advanced fabrication strategies, including electron-beam lithography, nanoimprinting, high-resolution printing/writing, and emerging device architectures like vertical OECTs and internal ion-gated OECTs, offer promising solutions (*51*, *52*). Vertical OECTs, for example, have achieved impressive densities of ~7.2 million OECTs/cm^2^ with response times in the microsecond range (*53*) and have been successfully integrated into front-end amplifier circuits for flexible neural implants (*18*).

In parallel, organic semiconductor–based artificial synapses have made notable strides in scalability and reliability (*54*, *55*), offering promise for future integration with d-OECNs to create efficient neural microcircuits for edge processing. We demonstrate a preliminary simulation of this integration in figs. S17 and S18. By leveraging the capabilities of d-OECNs, we can emulate biological sensory processing more efficiently, leading to simpler architectures for event-based sensors and improved feature extraction at the sensor level by augmenting the functionality of silicon-based neuromorphic processors. In addition to such applications, the study also encourages further exploration into multicompartmental neuron models using OECTs to emulate various neuron sections, including the potential for mimicking additional dendritic functionalities, such as spatial-temporal summation. These prospects underscore the wide range of opportunities for advancing our understanding and utilization of OECNs in mimicking biological neurons and networks.

## MATERIALS AND METHODS

### Materials

Naphthalenetetracarboxylic dianhydride, 1,2,4,5-tetraaminobenzene tetrahydrochloride, poly(phosphoric acid), methanesulfonic acid (MSA), chloroform, 1,2-dichlorobenzene, ethylene glycol, (3-glycidyloxypropyl)trimethoxysilane, and 4-dodecylbenzenesulfonic acid were sourced from Sigma-Aldrich. BBL, with a viscosity of 6.3 dl g^−1^ in MSA at 30°C and a molecular weight of 35 kDa, was synthesized through the polycondensation of naphthalenetetracarboxylic dianhydride and 1,2,4,5-tetraaminobenzene tetrahydrochloride in poly(phosphoric acid) under high-temperature conditions (*56*, *57*).

### Thin-film casting

BBL is initially dissolved in MSA and heated at 100°C for 12 hours. After cooling to ambient temperature, the resulting BBL-MSA solution is applied to OECT substrates using a spin-coating process (1000 rpm for 60 s, with an acceleration of 1000 rpm s^−1^). Any remaining MSA in the film is then removed by soaking in deionized water, followed by drying under a nitrogen stream.

### OECT fabrication and testing

The fabrication and testing of OECTs follow an established method, as detailed in previous works (*56*, *58*). The process begins with a thorough cleaning of 4-inch (10.16-cm) glass wafers using acetone, deionized water, and isopropyl alcohol, followed by drying with nitrogen. The electrodes, comprising 5 nm of chromium and 50 nm of gold, are deposited by thermal evaporation and patterned using photolithography. A 1-μm parylene C layer is applied over the electrodes in the presence of 3-(trimethoxysilyl)propyl methacrylate (A-174 Silane) (to enhance adhesion) to serve as an insulating barrier, reducing capacitive interference at the electrode-electrolyte boundary. Subsequently, a 2% Micro-90 industrial surfactant is spin coated as an antiadhesive layer. Over this, a sacrificial layer of parylene C (2 μm thick) is deposited, followed by a 5-μm-thick layer of AZ10XT520CP positive photoresist. This setup protects the underlying layers during the plasma reactive ion etching step [150 W, O_2_ = 500 SCCM (standard cubic centimeter per minute), CF_4_ = 100 SCCM, for 380 s]. Photolithography is again used to delineate the contact pads and the OECT channel, after which the photoresist is developed with AZ Developer. The plasma reactive ion etching step then removes the photoresist and sacrificial parylene, exposing the OECT channel and contact pads while preserving the other areas with parylene layers. The channels are patterned to specific width/length ratios: 80 μm/6 μm for Na-OECTs. The BBL-MSA solution is then spin coated to achieve a film thickness of 20 nm (Na-OECT) (fig. S19). The sacrificial parylene layer is peeled off, leaving behind a semiconductor film confined to the wells and connecting the source/drain electrodes of the OECT. Ag/AgCl paste is used to form a gate electrode (1 μm thick, 9 mm^2^). All OECT measurements are conducted in a 0.1 M NaCl aqueous solution, unless specified otherwise. The devices are characterized using a Keithley 4200A-SCS.

### SPICE simulations

The SPICE models of the d-OECT and d-OECN were developed using B2 SPICE (EMAG Technologies). These models are designed to simulate the spiking behavior of the d-OECN as well as the modulation of decision boundaries in XOR classifications. Detailed descriptions of the simulation setup and results are provided in the Supplementary Materials.
