# Supplementary material for: Single organic electrochemical neuron capable of anticoincidence detection
Source: Sci Adv. 2025 Jun 20;11(25):eadv3194. doi: 10.1126/sciadv.adv3194 (PMC12180488; doi:10.1126/sciadv.adv3194)
Supplement: Supplementary file 1 — Supplementary Text Figs. S1 to S19 [file sciadv.adv3194_sm.pdf]

Science Advances
AAAS

Supplementary Materials for

Single organic electrochemical neuron capable of anticoincidence detection

Padinhare Cholakkal Harikesh *et al.*

Corresponding author: Padinhare Cholakkal Harikesh, harikeshpc@riseup.net;
Simone Fabiano, simone.fabiano@liu.se

Sci. Adv. **11**, eadv3194 (2025)
DOI: 10.1126/sciadv.adv3194

This PDF file includes:

Supplementary Text
Figs. S1 to S19

Supplementary Text

Note S1: Operation of the soma part of the circuit.

The operation of the soma part in the *d*-OECN circuit utilizes a conductance-based model, incorporating sodium and potassium channels that mimic the Hodgkin-Huxley (HH) model. The circuit comprises two main transistors: a sodium ion-based transistor (Na-OECT) and an NMOS transistor (T_K), each connected to two distinct voltage sources, E_{Na} (500 mV) and E_K (ranging from ~0.71 to -0.75 V). These components parallel the ion channels and batteries in the original HH model, as illustrated in Fig. S1. Voltage E_{Na} is applied to the drain of Na-OECT and E_K to the source of T_K.

In this setup, an input current I_{in}, varying between 2-15 μA, is fed into the circuit. This current is accumulated by the membrane capacitance C_{mem}, causing the membrane potential V_{mem} to rise from its baseline. Concurrently, this rise in potential drives the gate voltage of Na-OECT from approximately 1.2 V to 0.8 V via an n-type metal-oxide-semiconductor (NMOS) based inverting amplifier (Fig. S1D). This adjustment enables the device to navigate through the peak of the antiambipolar transfer curve, initiating a spike in the current, which further charges the capacitor, thereby sharply increasing V_{mem} (depolarization phase).

Following this, the NMOS transistor T_K activates after a brief delay, controlled by resistor R_K and capacitor C_K, and achieves its maximum current subsequent to the peak current of Na-OECT. The capacitor discharges through T_K, leading to a decrease in V_{mem} (repolarization phase) and returning it to its original level. Due to the higher and longer-lasting current of the K-OECT, the voltage momentarily dips below the baseline (hyperpolarization phase). This sequence of events recurs cyclically with a constant input current, thereby sustaining the generation of action potentials. Adjustments to the threshold T_K can be made by modifying E_K from approximately -0.71 to -0.75 V, optimizing the operational range of the soma necessary for effective integration within the *d*-OECN system.

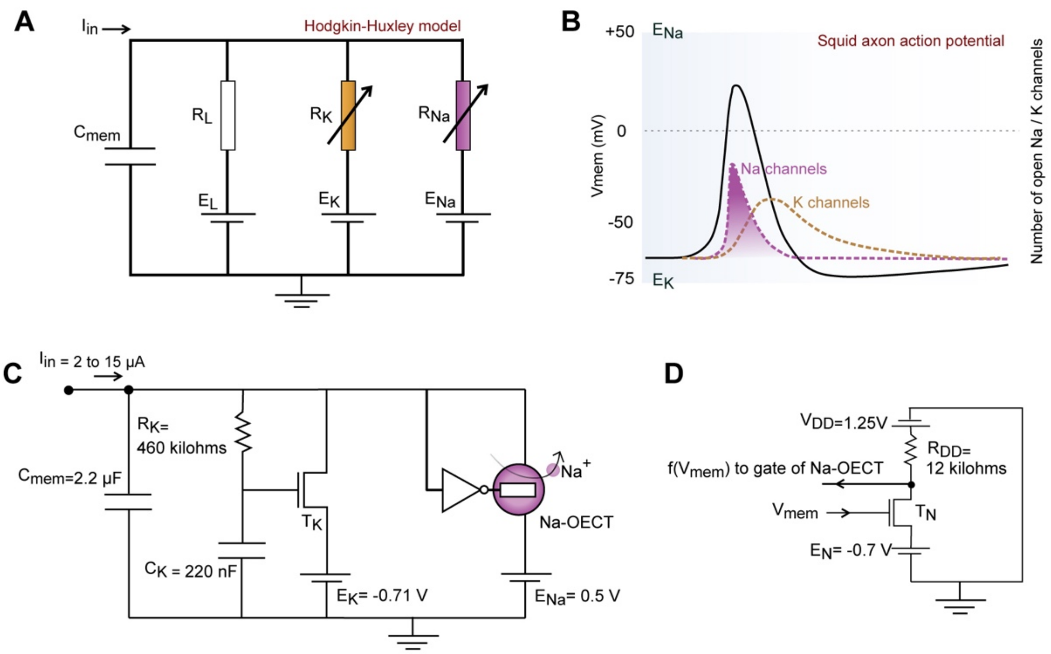

Fig. S1. Hodgkin-Huxley (HH) neuron model and circuit implementation. A) Schematic of the HH neuron model depicting sodium (Na^+) and potassium (K^+) ion channels. **B**) Graphical representation of a biological action potential with the activity profiles of Na^+ and K^+ ion channels. **C**) The c-OECN (soma) circuit with corresponding components. **D**) Detailed circuit diagram of the NMOS-based inverting amplifier utilized within the soma circuit. Both T_K and T_N are NMOS transistors (Infineon BSP295 model). Components R_K and C_K introduce the necessary delay for emulating the dynamics of the potassium channel.

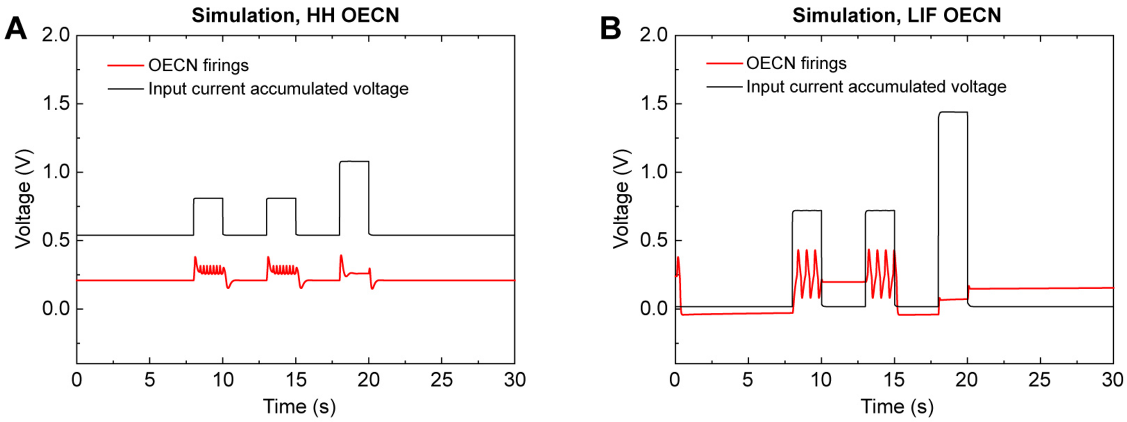

Fig. S2. SPICE simulation of XOR spiking behavior in *d*-OECNs. Simulated XOR spiking behavior of the *d*-OECN model with (**A**) an HH and (**B**) a LIF neuron. In the SPICE simulation, the *d*-OECN circuits consist of a *d*-OECT with a voltage-integrating resistor at the gate, paired with either the HH or LIF OECN.

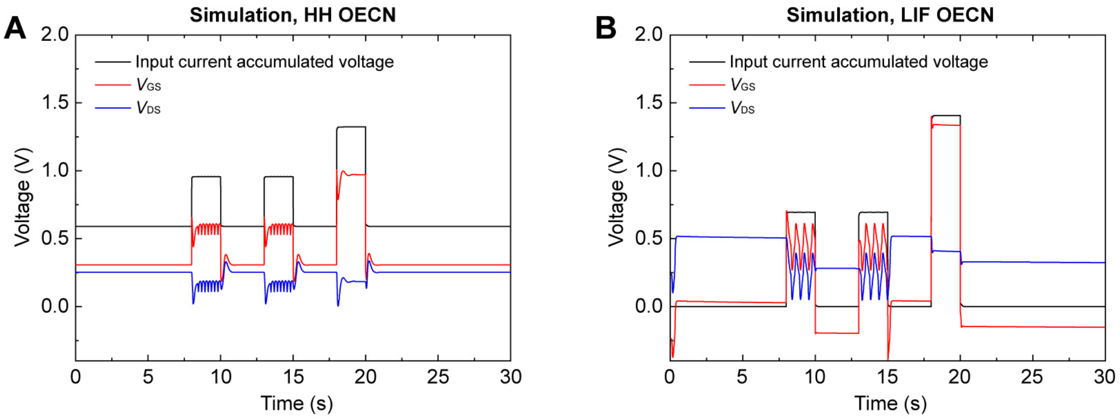

Fig. S3. Effective V_{GS} and V_{DS} in *d*-OECNs. Simulated V_{GS} and V_{DS} at different operational states of (**A**) an HH *d*-OECN and (**B**) a LIF *d*-OECN. The source terminal of the *d*-OECT is connected to the V_{mem} node. Therefore, the effective V_{GS} and V_{DS} have different baselines under different input conditions and would fluctuate when the neuron spikes.

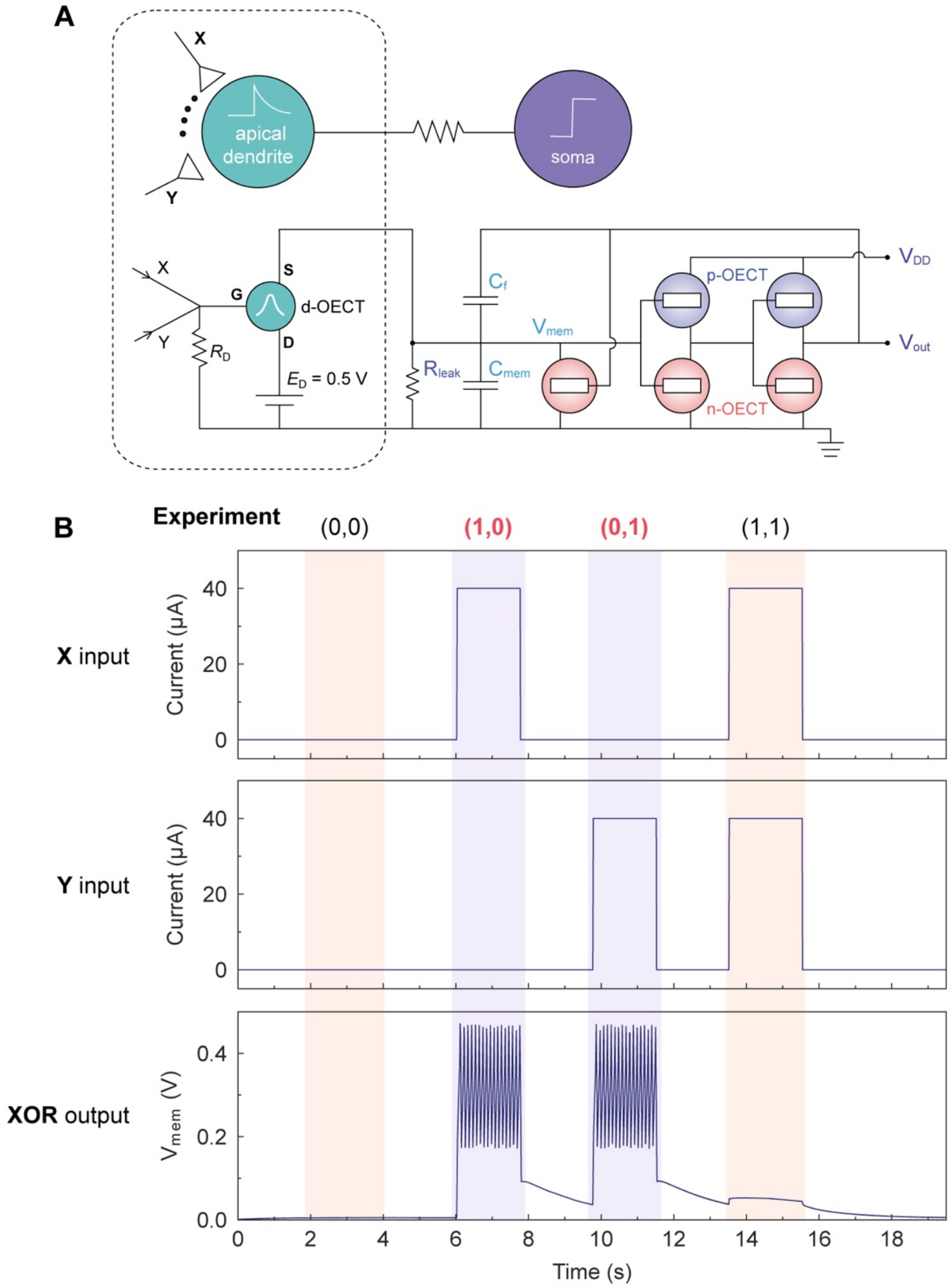

Fig. S4. *d*-OECN incorporating a leaky integrate-and-fire (LIF) soma. A) Schematic circuitry layout of the *d*-OECN incorporating a LIF-OECN as the soma. Circuit parameters are as follows: R_D = 18 kilohms, E_D = 0.5 V, R_{leak} = 100 kilohms, $C_f = C_{mem}$ = 1 μF, V_{DD} = 0.6 V. **B**) Experimental XOR response of the *d*-OECN integrated with a LIF-OECN-soma. R_{leak} represents the intrinsic leaky component in the LIF model. In this implementation, the inclusion of R_{leak} serves to set the current threshold above which the neuron begins to spike. The neuron is thereby designed to spike only when the *d*-OECT supplies a high current near its Gaussian peak to enable XOR, with a lower R_{leak} (higher leakage) yielding a narrower XOR boundary. This strategy differs from that employed in the HH model, where the bias on K-OECT (E_K) controls the neuron's leaky characteristics, spiking threshold, and XOR boundary.

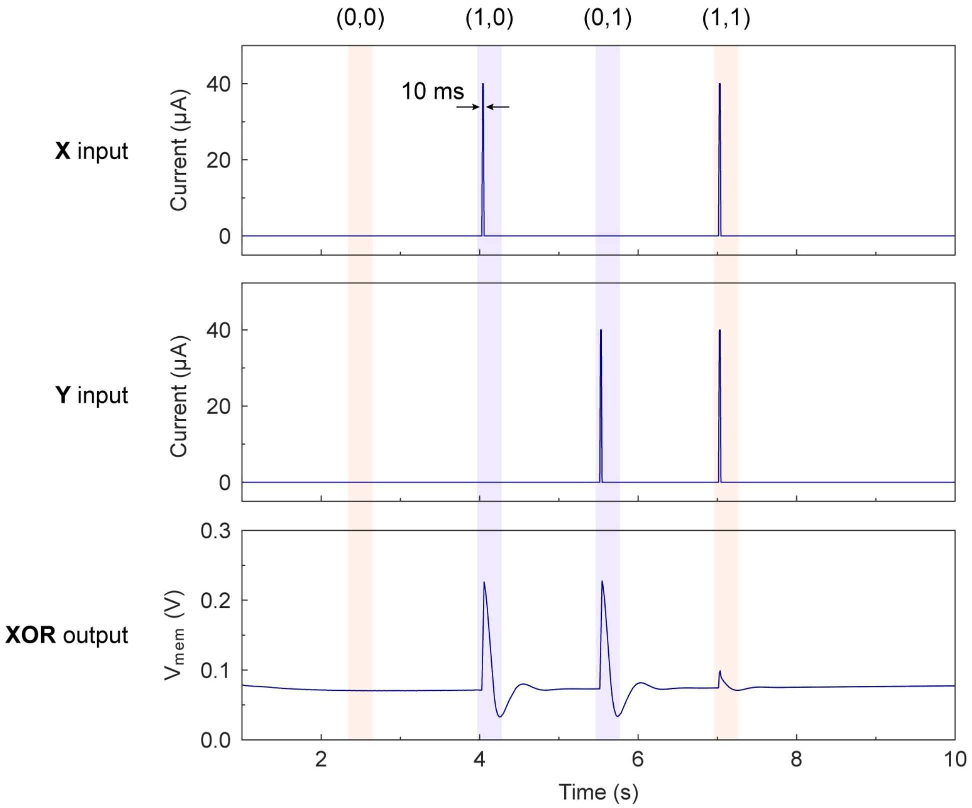

Fig. S5. Spiking response to short-duration X/Y inputs. Response of the *d*-OECN to 10-ms X/Y current inputs.

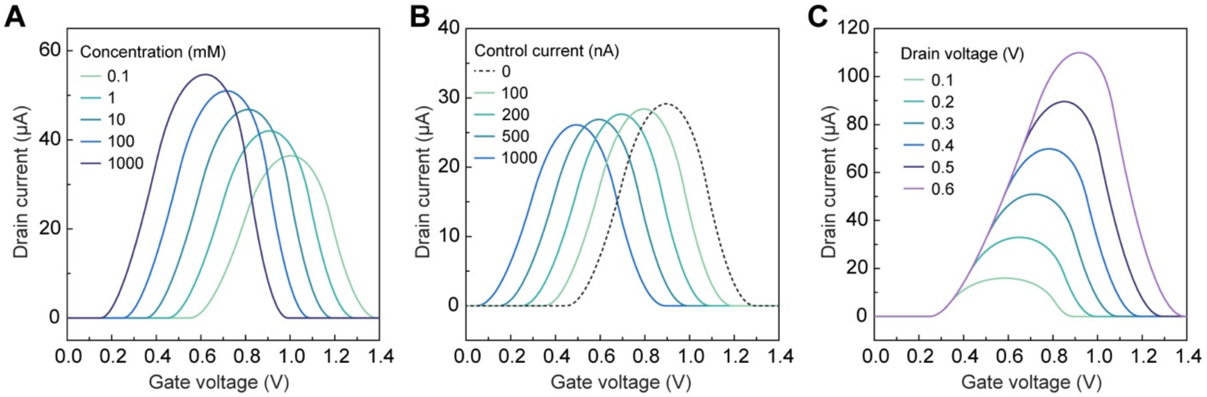

Fig. S6. Simulated *d*-OECT transfer characteristics. Simulated transfer characteristics of *d*-OECTs at varying ionic concentrations (**A**), control currents in a dual gate configuration (**B**), and drain voltages (**C**). The *d*-OECT SPICE model used for these simulations is based on equivalent circuits and represents an advanced update of the previously reported antiambipolar BBL-based OECT model (see Ref. (*6*) in the main text).

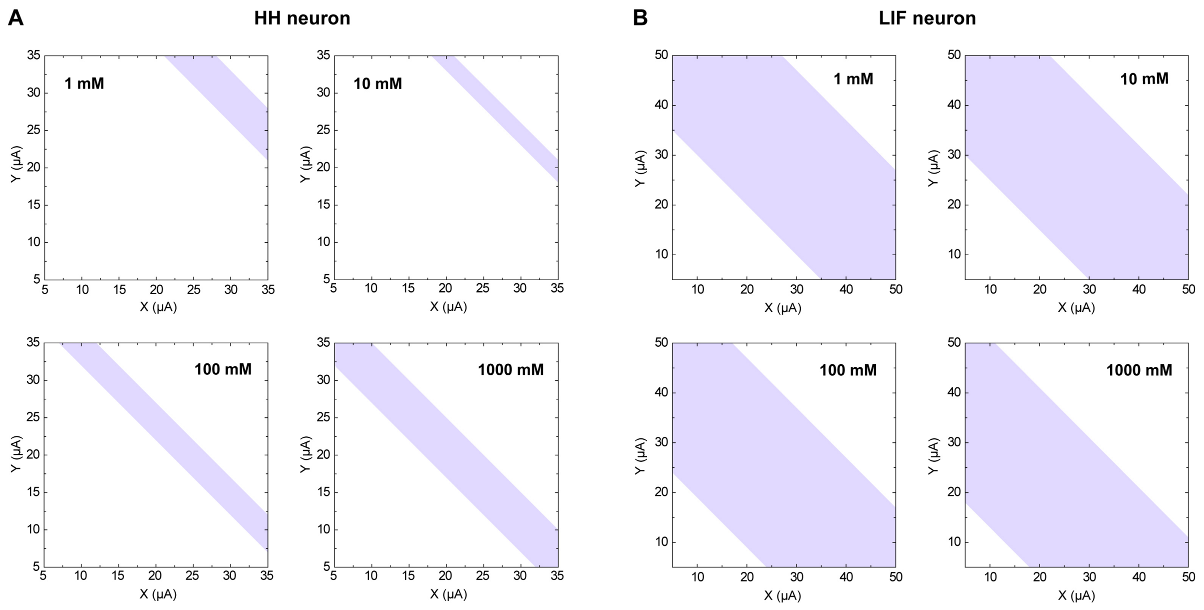

Fig. S7. Simulated decision boundaries. Simulated decision boundaries of the *d*-OECN using an HH neuron model (A) or a LIF neuron model (B) as a function of the ionic concentration.

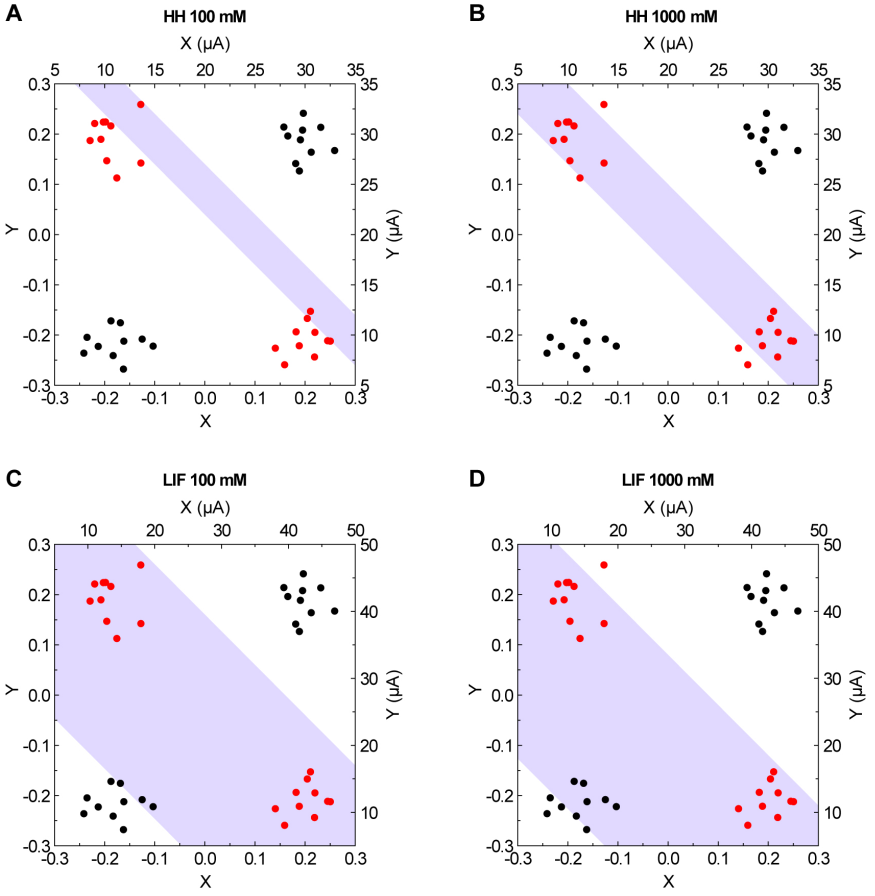

Fig. S8. Simulated XOR classification. Simulated XOR classification for a randomly generated dataset as a function of the ionic concentration for the HH *d*-OECN (**A**-**B**) and LIF *d*-OECN (**C**-**D**). In the simulation, the dataset $X \sim N(\mu, 0)$ consists of two clusters, each with two data points. The cluster means are $\mu_1 = (-0.2, 0.2)$ and $\mu_2 = (0.2, -0.2)$, representing class 1 (red) and 0 (black), respectively. The X, Y input currents are mapped to this dataset.

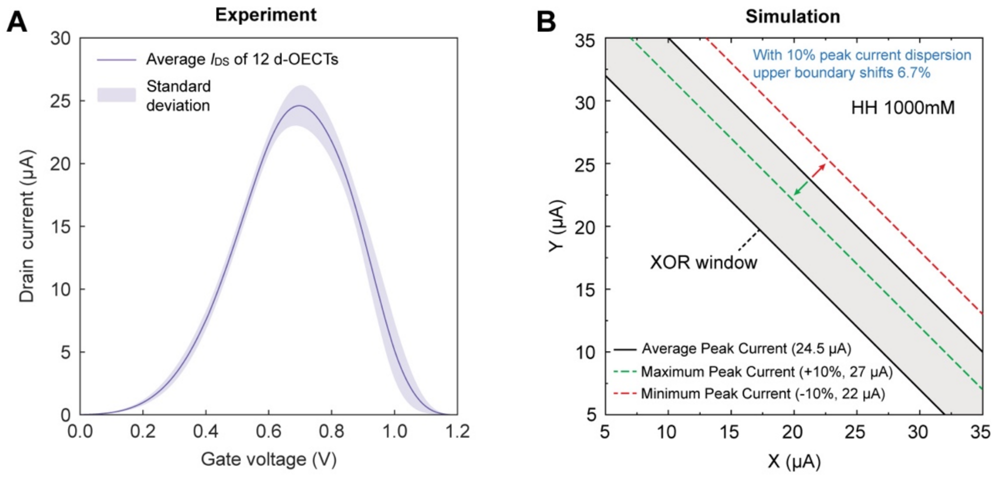

Fig. S9. Impact of device variability on decision boundaries. A) Transfer characteristics of 12 BBL-based *d*-OECTs (W/L = 100 μm/10 μm, $I_{D,peak}$ = 24.5 ± 2.5 μA), demonstrating a device-to-device current variability of approximately 10%, typical for photolithographically-made devices. **B**) Simulated decision boundaries as a function of peak current variation. The simulation indicates that the upper decision boundary shifts by ~6%, while the lower decision boundary is unaffected. This difference arises because current variability is considerably smaller at lower gate voltages than at higher gate voltages, resulting in a more pronounced shift in the upper decision boundary.

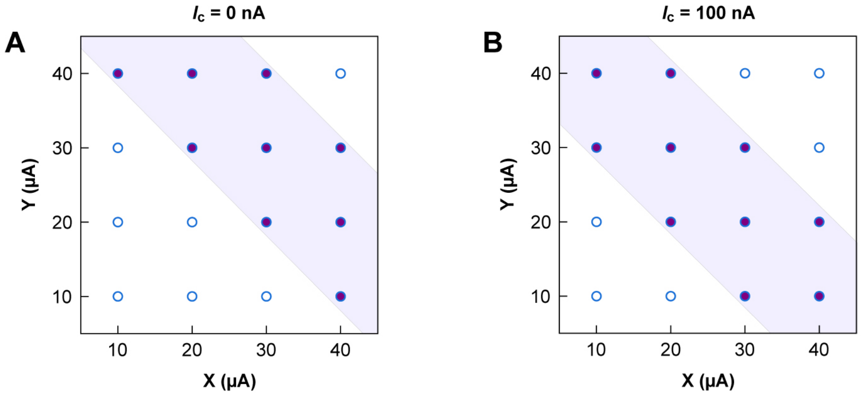

Fig. S10. Control current modulation of XOR decision boundaries. Modulation of the XOR decision boundaries by injecting a control current to the secondary gate of the *d*-OECT. Decision boundary with I_C = 0 nA (**A**) and I_C = 100 nA (**B**).

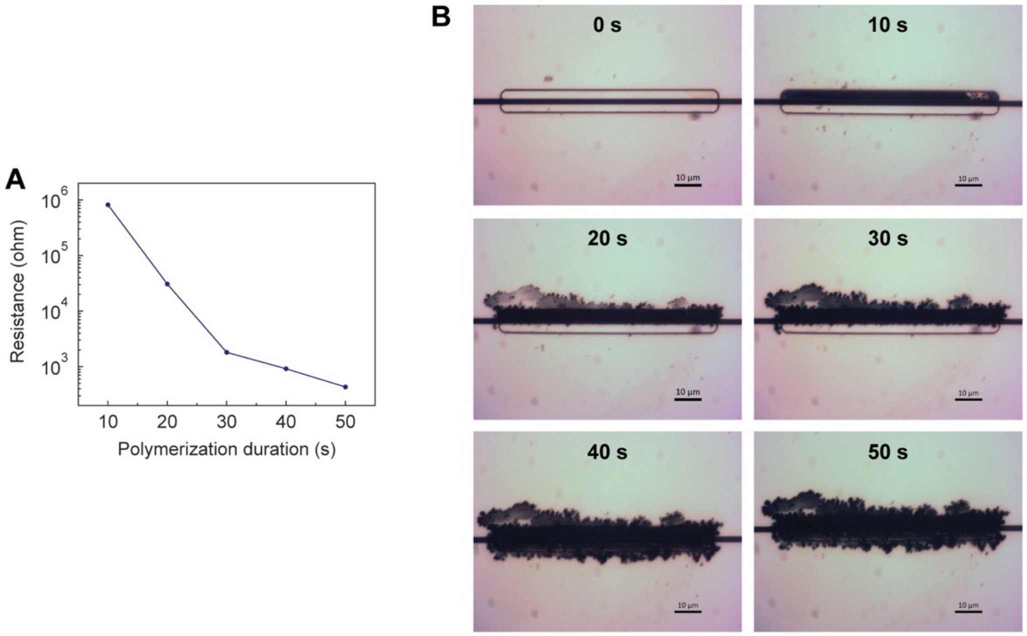

Fig. S11. Electropolymerized organic electrochemical synapses. A) Resistance changes in an organic electrochemical synapse as a function of varying electropolymerization periods. **B**) Microscopic images showing the progressive deposition of channel materials corresponding to increasing electropolymerization durations. Synapse growth is achieved by electropolymerizing sodium 4-(2-(2,5-bis(2,3-dihydrothieno[3,4-b][1,4]dioxin-5-yl)thiophen-3-yl)ethoxy)butane-1-sulfonate (ETE-S) across the channel area of an OECT (see Ref. (*11*) in the main text). The channel is 80 μm wide and 6 μm long. A drop of ETE-S monomer solution is applied to cover the channel area while in contact with an Ag/AgCl pellet gate electrode. During electropolymerization, the gate is biased at -0.6 V while the drain and source are grounded.

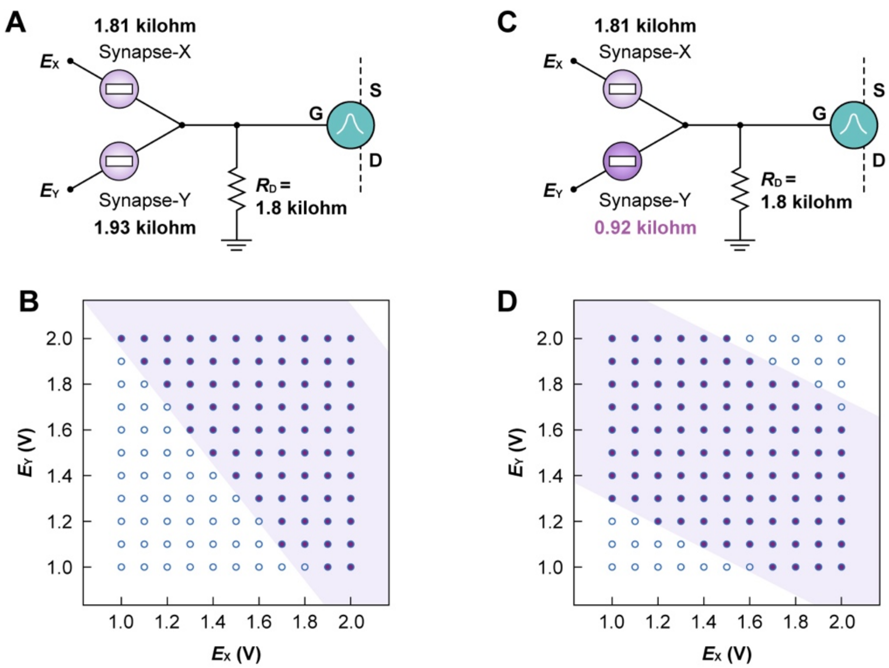

Fig. S12. *d*-OECT integrating organic electrochemical synapses. **A**) Schematic circuit of the *d*-OECT integrating two organic electrochemical synapses as X/Y inputs, with both synapses electropolymerized for 30 seconds. **B**) Decision boundary of the *d*-OECN integrating two synapses (each with a 30-second electropolymerization period) as X/Y inputs. **C**) Configuration where the electropolymerization period for synapse-X remains at 30 seconds, while that for synapse-Y is increased to 40 seconds. **D**) Decision boundary of the *d*-OECN integrating the modified synapses (30 seconds for synapse-X, 40 seconds for synapse-Y) as X/Y inputs, illustrating the influence of synaptic properties on classification performance. During XOR operation, E_X and E_Y are applied as drain voltages to the synapses, which operate as two-terminal resistive elements in this testing configuration—requiring neither a gate electrode nor the electrolyte. The synapse weight (*i.e.*, conductance) is modulated via the electropolymerization process shown in Fig. S11, during which an Ag/AgCl gate electrode is temporarily introduced to assist the reaction.

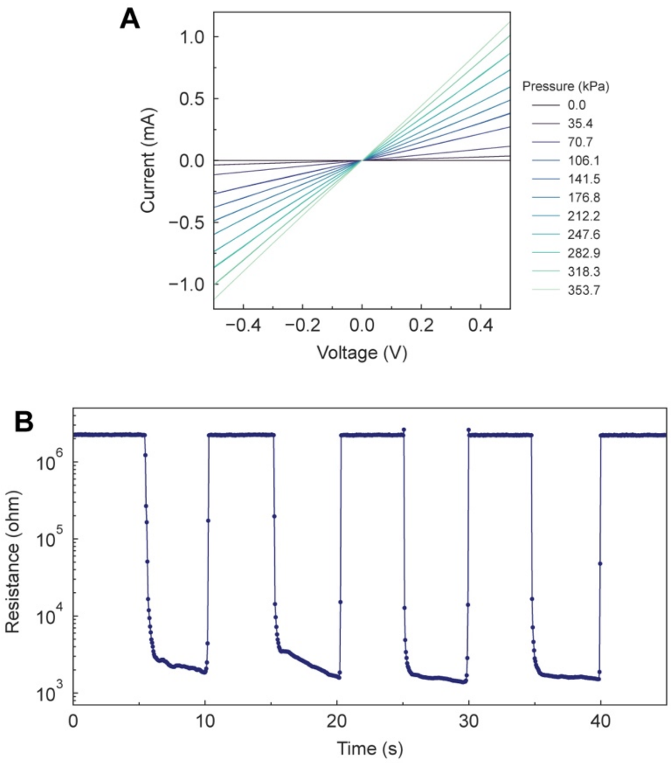

Fig. S13. Electrical characteristics of the pressure sensor. A) I-V characteristics of the pressure sensor under different pressure inputs. **B**) Continuous recording of the pressure sensor's resistance changes in response to 4 consecutive pressing inputs applied using a finger.

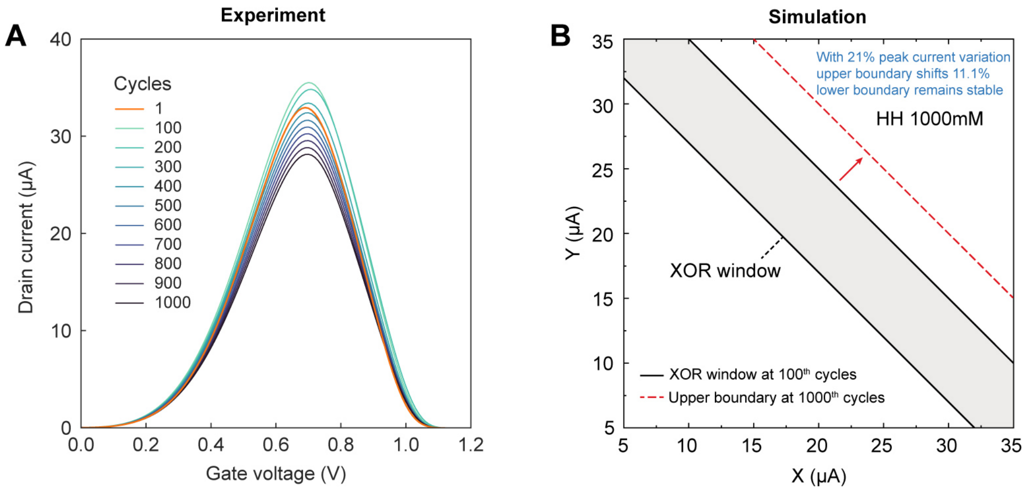

Fig. S14. Stability of *d*-OECTs with Ag/AgCl gate. A) Transfer curves of the first and every 100th cycle. The peak current increased from 32.9 μA to 35.5 μA during the first 100 cycles, then gradually declined to 27.4 μA at the 1000th cycle. The peak position remains stable. **B**) Simulated decision boundaries as a function of peak current variation. The results suggest that a 21% variation in peak current causes an 11.1% shift in the upper boundary while the lower boundary is stable.

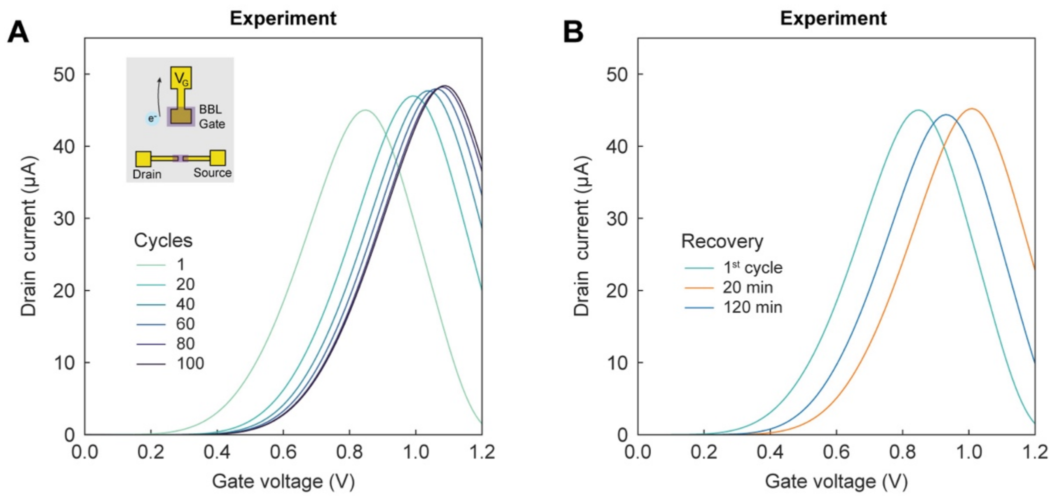

Fig. S15. Stability of *d*-OECTs with BBL gate. A) Transfer curves of the first and every 20th cycle. **B**) Recovery of peak position after 20 and 120 minutes, respectively. The gate, source, and drain electrodes were left open-circuit in between measurements throughout the recovery process.

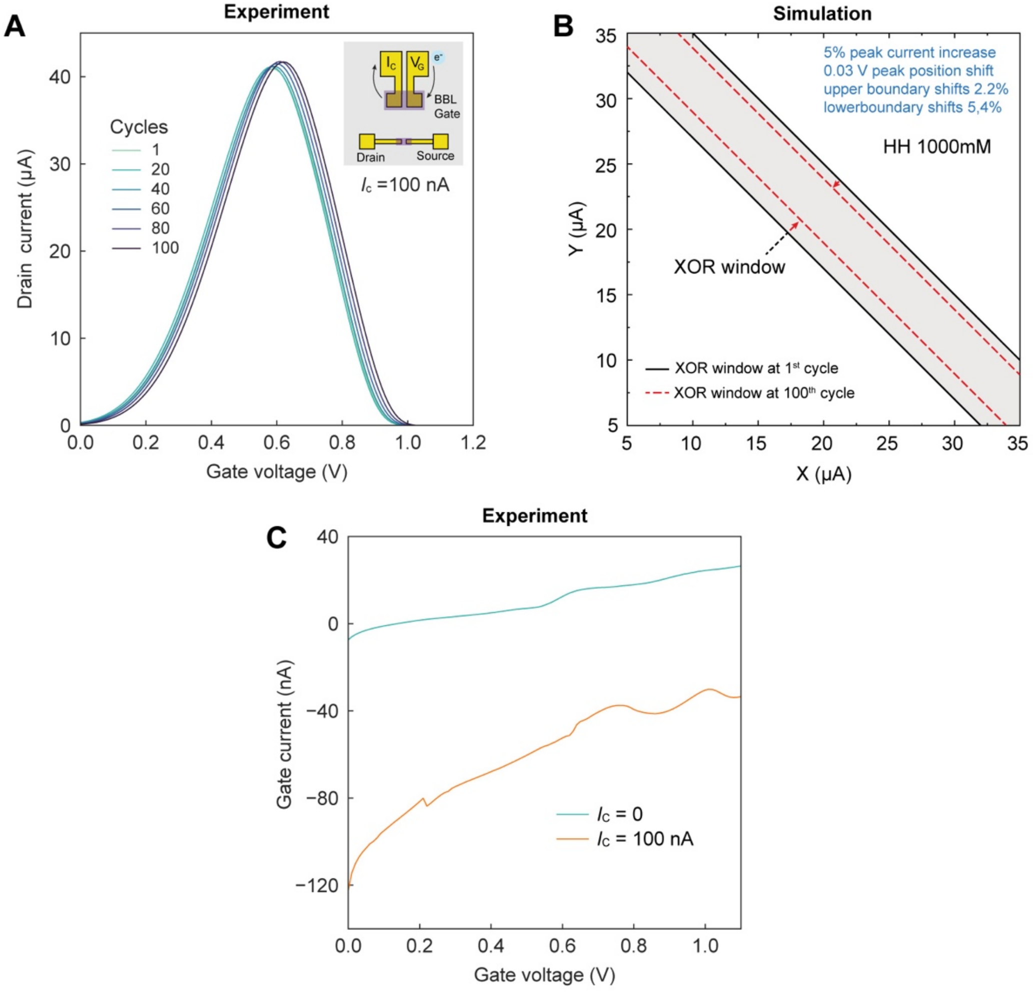

Fig. S16. Stability of *d*-OECTs with dual-gate design. A) Transfer curves of the first and every 20th cycle. The peak current increased by 5% while the peak position shifted 0.03 V higher. **B**) Simulated decision boundaries as a function of peak current variation. The simulation results suggest that the variations at the 100th cycle showed a 2.2% shift of the upper boundary and a 5.4% shift of the lower boundary. **C**) Recorded gate current under different I_C biases. The I_C injection reverses the direction of electron flow in the V_G electrode, bringing electrons from gold into BBL (reduction). This method stabilizes the BBL on the V_G electrode.

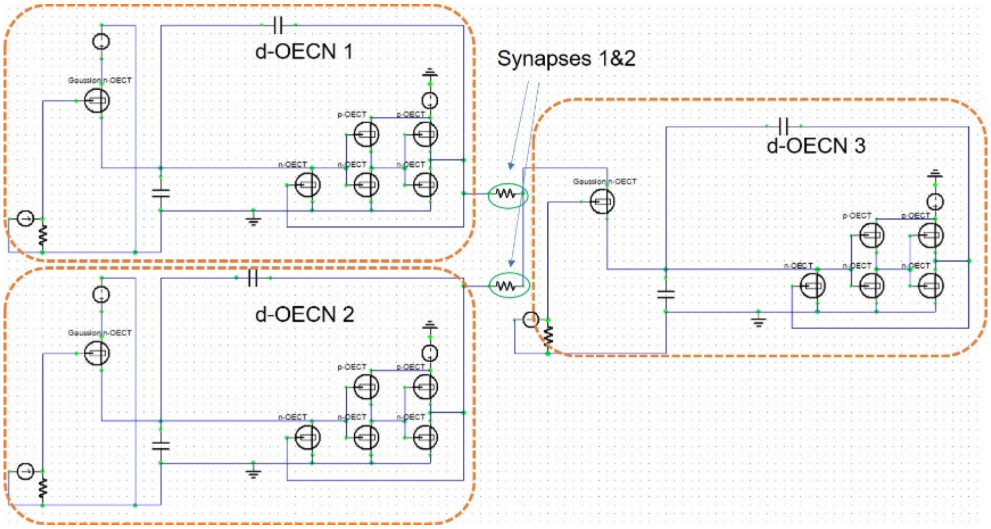

Fig. S17. SPICE simulation of a neural network with *d*-OECNs and synapses. *d*-OECN 1 and 2 act as presynaptic neurons, and their firings accumulate through synapses 1 and 2 into *d*-OECN 3. The activation of the *d*-OECNs is controlled by the incoming current, which accumulates as gate voltage on the *d*-OECTs. This shows how the circuit can be integrated into larger computing systems. The accumulated gate voltages of all *d*-OECNs are reported in Fig. S18.

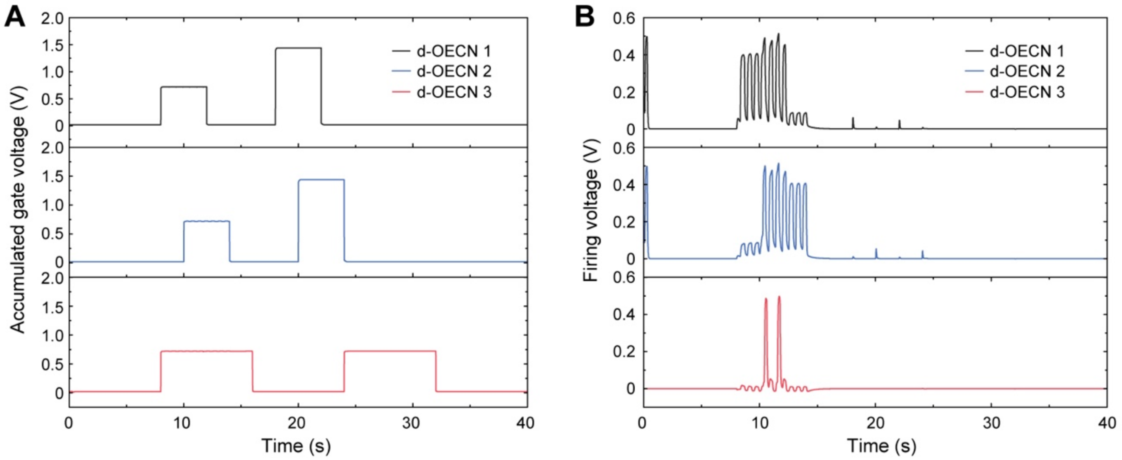

Fig. S18. Simulated spiking behavior of the network. Accumulated gate (**A**) and firing voltages (**B**). *d*-OECN 3 spikes only when both *d*-OECN 1 and 2 are firing and at a lower frequency. This demonstrates the potential for integrating *d*-OECNs and synapses into larger networks for more complex processing and computing tasks.

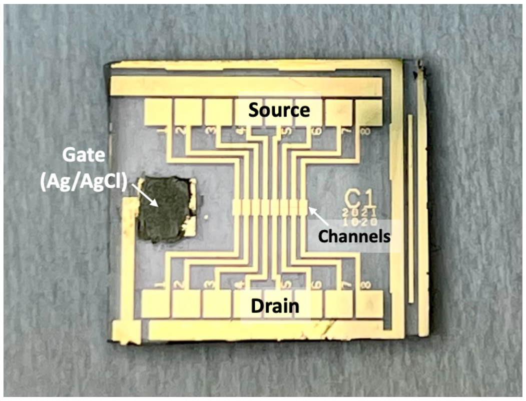

Fig. S19. Schematic of the OECT architecture. Structure of the OECTs used in this study.
